# Results after one year of rotavirus universal mass vaccination in Sicily

**DOI:** 10.1186/1824-7288-41-S2-A77

**Published:** 2015-09-30

**Authors:** Francesco Vitale, Fabio Tramuto, Emanuele Amodio, Vincenzo Restivo, Claudio Costantino

**Affiliations:** 1 Department of Science for Health Promotion and Mother to Child Care “G. D'Alessandro”, University of Palermo, Palermo, Italy

## Background

Rotavirus (RV) vaccination is the best strategy to prevent hospitalizations due to rotavirus gastroenteritis (RVGE) and is strongly recommended by international health authority [1]. The Sicilian Health Department introduced rotavirus universal mass vaccination (RUMV) into regional immunizations schedule in 2013 (mean vaccination coverage = 31%).

Intussusception is the invagination of one segment of the intestine within a more distal segment and even though the etiology is still unknown, in 1998, a relationship with a tetravalent rotavirus vaccine that was promptly withdrawn was suggested [2]. Post licensure surveillance studies have not confirmed previous findings and no increased risk of intussusception was found between vaccinated infants with both of the actually licensed rotavirus vaccines [3,4].

Aim of this study is to analyze the trend of RVGE hospitalizations and contextually to monitor the trend of intussusception in Sicily from 2009 to 2013 after one year of RUMV.

## Material and methods

Were collected data from hospital discharge records occurred from 1^st^ January 2009 to 31^st^ December 2013 in Sicily.

Cases of RVGE were defined as all hospitalizations with an ICD-9-CM diagnosis code of 008.61 on any position [5]. Furthermore, cases of intussusception were defined as all hospitalizations with an ICD-9-CM code of 560.0 on any discharge diagnoses.

## Results

**Figure 1:**
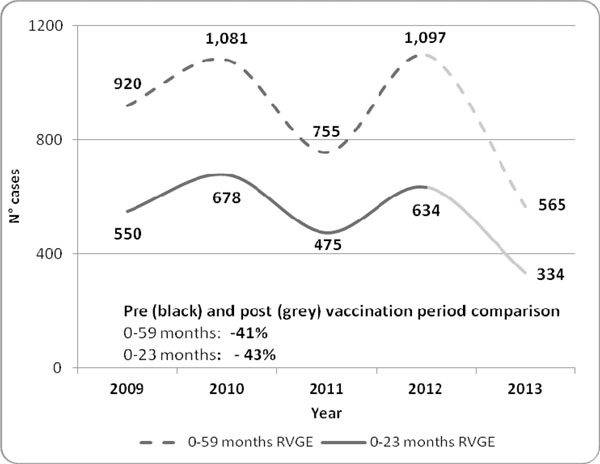
Hospitalization cases for RVGE in children aged 0-59 and 0-23 months in Sicily from 2009 to 2013.

**Figure 2:**
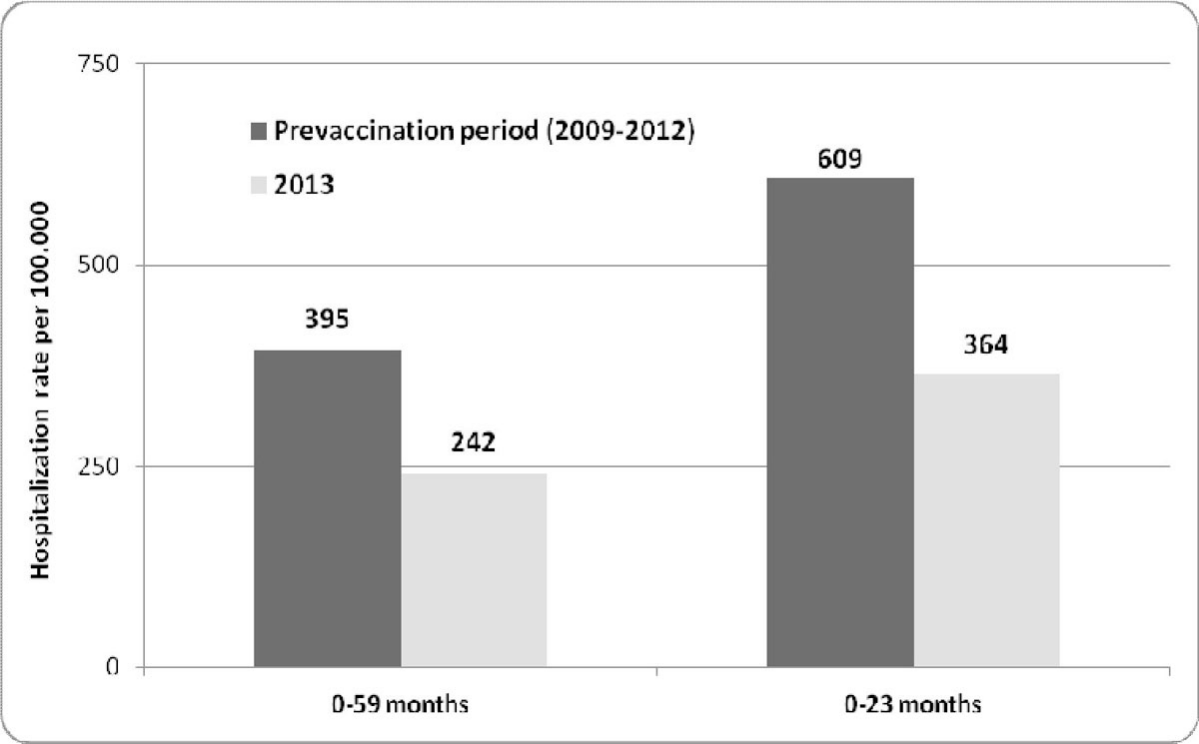
Hospitalization rates fro GERV observed before (2009-2012) and after (2013) RV vaccination introduction in Sicily (age classes 0-59 months and 0-23 months)

In 2013 the RVGE hospitalizations were 41% less in children aged 0-59 months and 43% less in children aged 0-23 months respect to the mean number of cases observed from 2009 to 2012 in Sicily (figure 1). Analyzing RVGE hospitalization rates per 100,000, was reported a significant reduction in both age classes in 2013 respect to mean incidence observed from 2009 to 2012 (0-59 months: from 395 to 242 cases/100,000; 0- 23 months from 609 to 364 cases/100,000) (figure 2).

**Figure 3:**
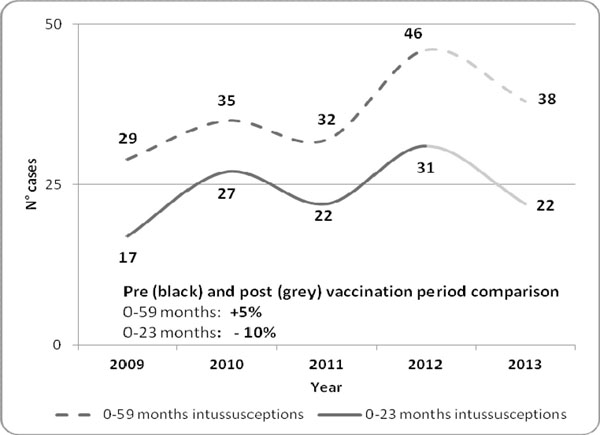
Hospitalization cases for intussusception in children aged 0-59 and 0-23 months in Sicily from 2009 to 2013

Finally, a significant increase in intussusception hospitalizations was not reported with respect to mean number of hospitalized children observed from 2009 to 2012 in age class 0 to 59 months. In particular, among children aged 0-23 months (directly exposed to vaccination in 2013) a slight reduction was observed with respect to mean number of cases reported in 2009-2012. (figure 3).

## Conclusions

After one year of surveillance and despite reaching low vaccination coverage, our results demonstrated the high effectiveness of the RUMV strategy on reduction of RV circulation. Similar data on RV vaccination efficacy on early vaccination campaign was reported in Belgium [6].

Moreover, the steadiness of intussusception hospitalizations after introduction of RV vaccination allows us to confirm the security profile of the available vaccine.
